# Solvent Treatment of Wet-Spun PEDOT: PSS Fibers for Fiber-Based Wearable pH Sensing

**DOI:** 10.3390/s19194213

**Published:** 2019-09-28

**Authors:** Daniel O. Reid, Rachel E. Smith, Jose Garcia-Torres, John F. Watts, Carol Crean

**Affiliations:** 1 Department of Chemistry, University of Surrey, Guildford GU2 7XH, UK; danieloliverreid@gmail.com (D.O.R.); Rachel.e.smith111@gmail.com (R.E.S.); garcia.torres.jm@gmail.com (J.G.-T.); 2 Department of Mechanical Engineering Sciences, University of Surrey, Guildford GU2 7XH, UK; J.Watts@surrey.ac.uk

**Keywords:** PEDOT: PSS, fiber electrode, wearable sensor, pH

## Abstract

There is a growing desire for wearable sensors in health applications. Fibers are inherently flexible and as such can be used as the electrodes of flexible sensors. Fiber-based electrodes are an ideal format to allow incorporation into fabrics and clothing and for use in wearable devices. Electrically conducting fibers were produced from a dispersion of poly (3,4-ethylenedioxythiophene)-poly (styrenesulfonate) (PEDOT: PSS). Fibers were wet spun from two PEDOT: PSS sources, in three fiber diameters. The effect of three different chemical treatments on the fibers were investigated and compared. Short 5 min treatment times with dimethyl sulfoxide (DMSO) on 20 μm fibers produced from Clevios PH1000 were found to produce the best overall treatment. Up to a six-fold increase in electrical conductivity was achieved, reaching 800 S cm^−1^, with no loss of mechanical strength (150 MPa). With a pH-sensitive polyaniline coating, these fibers displayed a Nernstian response across a pH range of 3.0 to 7.0, which covers the physiologically critical pH range for skin. These results provide opportunities for future wearable, fiber-based sensors including real-time, on-body pH sensing to monitor skin disease.

## 1. Introduction

Flexible and wearable electronics are being developed for use in a wide range of potential applications and is an active field of research [1,2,3,4]. True wearability requires such technologies to have fully flexible components, and fiber-based electrodes are an attractive option due to their electrically conductive and ductile nature. The fiber architecture allows for straightforward integration into textiles and garments making them comfortable to wear, with a “wear-and-forget” paradigm.

Electrically conducting fiber electrodes have been fabricated from carbons such as carbon nanotubes [5] and graphene [6] and conducting polymers such as polyaniline (Pani) [7], polypyrrole [8], and polyethylene dioxythiophene (PEDOT) [9]. Conducting polymers are particularly appealing for electronic textiles due to their intrinsic electrical and electrochemical properties such as high electrical conductivity, electrochemical switching, and charge storage [10,11,12,13]. The ease with which they can undergo solution processing allows continuous conductive fiber production using simple wet-spinning. Polyaniline (Pani) is interesting due to its variety of oxidation states that are pH dependent, making it suitable for pH sensing applications. Pani fibers have been produced by wet-spinning, yet are difficult to process due to its limited solubility [7]. Among conducting polymers, the highest electrical conductivity has been achieved with poly (3,4-ethylenedioxythiophene): Poly(4-styrenesulfonate) (PEDOT: PSS) after the removal of superfluous insulating PSS. Developed by Bayer in the late 1980s, PEDOT: PSS has good thermal and environmental stability and excellent processability from an aqueous dispersion, leading to its widespread use [14]. 

Studies have shown that solvent treatments for films of PEDOT: PSS have removed excess PSS and resulted in an increase in conductivity; however limited studies have been carried out using PEDOT: PSS fibers. Treatment with sulfuric acid has shown glass mounted PEDOT: PSS films to have a conductivity of over 3000 S cm^−1^ [15], while comparable treatments with formic acid displayed similarly high conductivities [16]. DMSO and methanol solvent treatment also greatly increased the conductivity of films [17,18]. Treatment of fibers with ethylene glycol showed a two-fold improvement in electrical conductivity in PEDOT: PSS fibers after one hour of immersion [19]. The modification of PEDOT: PSS to improve desirable properties is crucial to its use, due to the low electrical conductivity of water soluble PEDOT: PSS, typically in the order of 0.1–1.0 S cm^−1^ [19]. These improvements aid in increasing the performance and efficiency of devices. While PEDOT: PSS fibers have been produced [20] and used as strain sensors [21], limited examples exist describing their use in chemical sensing [22].

Achieving high electrical conductivities can allow PEDOT fibers to perform well as fiber electrodes. A highly conducting fiber is required for it to function as a base electrode during electrochemical deposition. The aim here was to investigate a broader range of solvent treatments on PSS fibers than has been carried out to date. The aim of these solvent treatments was to improve the fiber electrode properties, so that polyaniline could be electrodeposited onto these fibers for pH sensing applications. We have employed water soluble PEDOT: PSS pellets, and Clevios PH1000 PEDOT: PSS solutions to produce conducting PEDOT: PSS fibers. Treatment of these fibers after wet spinning was carried out via modification of the polymer using chemical treatments including sulfuric acid, formic acid, and dimethyl sulfoxide (DMSO). This allowed for excess insulating PSS to be removed, resulting in a vastly enhanced electrical conductivity at no expense to the mechanical properties. Subsequent electrochemical polymerisation of Pani onto PEDOT fiber electrodes allowed fiber-based pH sensing with a good Nernstian response.

## 2. Materials and Methods

PEDOT: PSS (Orgacon water soluble, re-dispersible pellets), acetone, iso-propanol, acetic acid, boric acid, phosphoric acid, nitric acid, hydrochloric acid, sulfuric acid, formic acid, DMSO, potassium chloride, and potassium ferricyanide were all purchased from Sigma Aldrich and used as received unless specifically stated. Heraeus Clevios PH1000 PEDOT: PSS was purchased at 1 wt.% from Heraeus. 

A 2 wt.% dispersion of Orgacon PEDOT: PSS was produced from the dried pellets. A 2 wt.% dispersion of Clevios PH1000 was prepared via evaporation of the as-received 1 wt.% solution at 50 °C. Coagulation was achieved via injection into a 50:50 mixture of acetone and iso-propanol rotating at 10 RPM. Needle diameters between 16 and 30 gauge with injection rates between 0.05 and 0.75 mL min^−1^ were used. Wet-spinning Clevios PH1000 PEDOT: PSS produced two fiber diameters of approximately 20 and 90 μm (20 microns; spun using a 30-gauge needle at 0.05 mL min^−1^ injection rate and 90 microns; spun using a 16-gauge needle at 0.75 mL min^−1^ injection rate), while the Orgacon PEDOT: PSS produced fiber diameters of 35 μm (using a 28 gauge needle with an injection rate of 0.75 mL min^−1^). The fibers were removed from the coagulation bath and dried under ambient conditions. Treatment of fibers was performed by immersion of dried fibers in solutions of sulfuric acid (aq, 1.5 mol dm^−1^), formic acid (aq, 25.2 mol dm^−1^), or DMSO (100% neat solvent). The fibers were immersed and left for 5, 60, and 120 min before being extracted and dried at 140 °C for 30 min. To construct pH-sensitive fibers, Pani was coated onto DMSO-treated fiber electrodes (1 cm length) using cyclic voltammetry between −0.2 to +1.0 V vs. Ag/AgCl for 10 cycles at 100 mV s^−1^ in solutions of aniline (0.1 M) in nitric acid (1.0 M). Potentiometry in Britton-Robinson pH buffered solutions was used for pH analysis and the potential of the Pani-PEDOT: PSS fiber electrodes (1 cm length) was measured against a Ag/AgCl double junction reference electrode (3.0 M NaCl internal solution, Alvatek, UK).

Raman spectra were recorded using an InVia Raman Microscope (Renishaw, Gloucestershire, UK) with a 532 nm laser; typically 1 mW laser power was employed. Conductivity measurements were taken using the four probe method with current applied using an eDAQ EA163 potentiostat and voltage measured using a Keithley 2001 multimeter. To ensure good contact, the fibers were mounted using silver paint across four electrical pins of equal spacing of 2 mm apart. Five different sections of fiber were measured and the average electrical conductivity is reported. Electrochemical depositions were performed using a Gamry Reference 600 potentiostat and Ag/AgCl reference electrode with platinum mesh counter electrode. Dynamic mechanical analysis was performed using a TA Instruments Q800 instrument. Scanning electron microscopy images were obtained using a JEOL USA JSM-7100F Analytical Field Emission SEM following gold coating of the samples. X-ray photoelectron spectroscopy (XPS) data was obtained using a ThermoFisher Scientific Theta Probe spectrometer with a monochromated Al Kα X-ray source. An X-ray spot of ~400 μm radius was employed in the acquisition of all spectra. Survey spectra were acquired employing a pass energy of 300 eV. High resolution, core level spectra for S2p were acquired with a pass energy of 50 eV.

## 3. Results and Discussion

Conducting polymer fibers can be produced by the readily scalable method of wet-spinning, which is capable of preparing kilometer-long conducting fibers. PEDOT: PSS fibers were produced by injecting 2.0 wt.% solutions of PEDOT: PSS into a coagulation bath consisting of acetone and iso-propanol (1:1 ratio). This system allowed meter-long quantities of fibers to be produced easily in a lab-scale set-up. Two commercially available sources of PEDOT: PSS were investigated including a solid pellet form (Orgacon) and a solution-based system (Clevios PH1000), with the latter yielding two different diameter fibers depending on the spinning injection rate. 

### 3.1. Effect of Solvent Treatment Time

**Figure 1:**
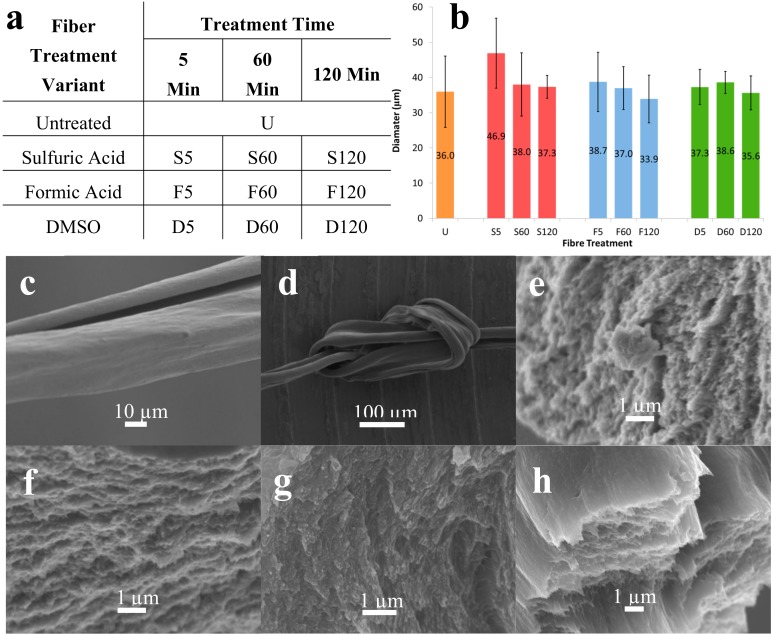
(**a**) Acronyms for Orgacon wet spun and chemically treated Orgacon PEDOT:PSS fibers, (**b**) average diameters of Orgacon fibers (n = 10), SEM images of: (**c**) fiber length of U, (**d**) dry knotted fiber U, (**e**) cross-section of U, (**f**) cross-section of S120, (**g**) cross-section of F120, (**h**) cross-section of D120.

Previous studies reported enhanced electrical conductivity of fibers following solvent treatment with ethylene glycol [19]. We extended the range of solvents to investigate the effect of sulfuric acid, formic acid, and dimethyl sulfoxide treatments on fiber properties, following reports that these solvents were effective at improving the electrical properties of PEDOT: PSS films [15,16,17]. The fibers were immersed into solutions of sulfuric acid, formic acid, and dimethyl sulfoxide for periods of 5, 60, and 120 min, followed by oven drying at 140 °C. Figure 1a describes the nomenclature used to name the fibers before and after solvent treatment. Initially, Orgacon PEDOT: PSS was used to study the effect of treatment time on fiber properties. No observable difference in diameter was observed following treatment with formic acid and DMSO, irrespective of treatment times, as shown in Figure 1d, and all fibers remained highly flexible, allowing them to be tied into knots. Alemu et al. reported a 5–10 nm change in film thickness following treatment with methanol as the solvent [18], which is difficult to see in fibers with micron-scale diameters such as ours. A 5 min treatment time with sulfuric acid increased the average diameter (although there is a 20% deviation within these measurements), which reduced again to those of untreated fibers for longer treatment times. This larger diameter is attributed to an increased water content of these fibers as observed during thermal gravimetric analysis (results not shown). We hypothesize that during the 5 min sulfuric acid treatment, sulfate ions enter the fiber (as observed during cyclic voltammetry), while PSS begins to diffuse out. The known strong hydrating effect of sulfate ions increases the water uptake in the fibers treated for 5 min, which leads to an increase in the diameter measured. Longer treatment times may allow the sulfate ion and PSS ion movement to reach an equilibrium and the fiber diameters are similar to those which have not been treated. SEM analysis of the cross-sectional area of untreated and treated fibers showed no apparent difference in morphology as a result of any of the treatments (Figure 1c–h). Optical microscopy determined the diameters of the Orgacon fibers to be in the range of 35 ± 10 µm.

**Figure 2:**
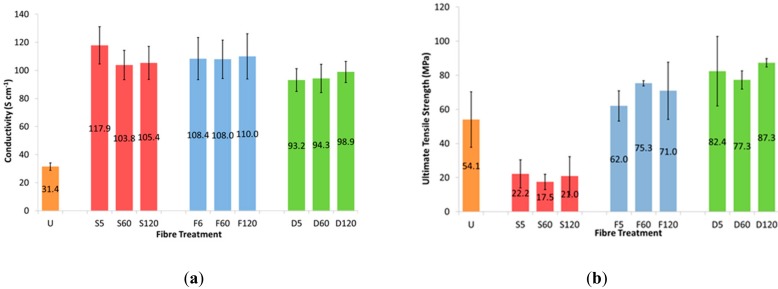
(**a**) Average conductivity measurements of untreated and solvent treated Orgacon PEDOT: PSS fibers (n = 5) and (**b**) average ultimate tensile strength values for treated Orgacon fibers (n = 5).

Electrical conductivity measurements of untreated and treated Orgacon fibers are shown in Figure 2. A threefold improvement in electrical conductivity was observed after just 5 min of solvent treatment. Similar conductivity values were measured for all fiber treatment times with no further improvement observed for longer times (either 60 or 120 min), suggesting that all modification of the fibers occurred within the first 5 min. Xia et al. found that the conductivity increased after treatment with dilute sulfuric acid for up to three treatments [13]. For the solvents used here, the treatments were repeated with multiple immersions, showing no further increase after the first submersion. A tripling of electrical conductivity values was observed after treatment, with little difference observed between the three solvents used. The average conductivity values were slightly higher for H_2_SO_4_ and formic acid treatments, when compared to treatment with DMSO. This trend however was not observed with other PEDOT: PSS sources (as shown later in Figure 4). This is partially attributed to the removal of insulating PSS from the fiber, specifically from the fiber surface and the reordering of the polymer chains and is discussed later in the sections dealing with Raman spectroscopy and XPS [7,13,15].

Similar mechanical properties were measured for all fiber treatment times, again suggesting that all modification of the fibers occurred within the first 5 min. Figure 2 shows the typical ultimate tensile strength values of both untreated and solvent treated Orgacon PEDOT: PSS fibers. Sulfuric acid treatments were found to significantly weaken the fibers leading to breaking strength values of less than half their untreated counterparts. There is no statistical difference in the breaking strengths of PEDOT: PSS fibers after treatment with formic acid and DMSO relative to untreated Orgacon fibers. The use of non-volatile DMSO as the treatment solvent followed by oven heating permits molecular re-orientation of the polymer chains into a stiffer fiber with a higher Young’s modulus compared to non-treated fibers, 2.0 GPa and 1.3 GPa, respectively (data not shown). DMSO and formic acid treatment improved the electrical/electrochemical properties of Orgacon PEDOT: PSS fibers at no detriment to their physical properties.

### 3.2. Effect of PEDOT: PSS Material Source

**Figure 3:**
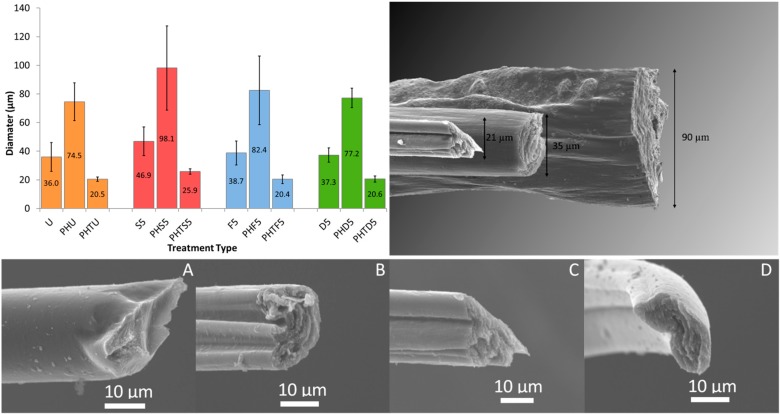
Averaged diameter values comparing Orgacon PEDOT:PSS fibers with both diameters of Clevios PH1000 fibers for 5 min treatments (**top left**) (n = 10). SEM graphic comparing the three fiber types (**top right**). SEM images of fiber cross-sections PHTU (**A**), PHTS5 (**B**), PHTF5 (**C**), PHTD5 (**D**).

The effect of solvent treatment on two different sources of PEDOT: PSS (Orgacon and Clevios PH1000) was also investigated. In total, three diameters of PEDOT: PSS fibers were studied and their nomenclature is described in Table 1. Wet-spinning Clevios PH1000 PEDOT: PSS produced two fiber diameters of approximately 20 and 90 µm, however it was only possible to spin Orgacon PEDOT: PSS fibers with diameters of 35 µm. Each fiber size was subjected to a 5 min treatment in sulfuric acid, formic acid, or DMSO; since it had been established that longer treatment times showed no extra benefit. More consistent diameters were produced for the smallest fibers spun from Clevios PH1000 PEDOT: PSS (PHTU; 20 µm), which was attributed to the faster coagulation time of thin fibers (Figure 3). SEM images of all three fiber types (Clevios; 20 µm, Orgacon; 35 µm and Clevios; 90 µm diameters) are shown in Figure 3, with no difference observed in the fiber cross-section or surface structure following treatment. Thin untreated fibers (PHTU) were found to display a smoother and more consistent overall surface profile, due to their faster coagulation time which generated fewer folds and bends before fiber solidification. No notable diameter change was observed following treatment for any of the fiber types (Figure 3).

**Figure 4:**
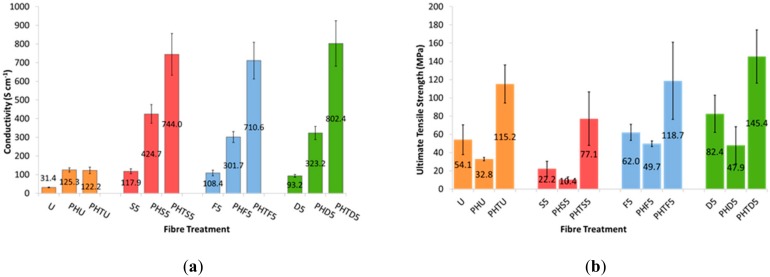
(**a**) Average electrical conductivity measurements and (**b**) average ultimate strength values for all untreated and all 5 min treated fibers; n = 5 in all cases (numbers on bars represent the average values).

Electrical conductivity values for all fiber types (untreated and treated for 5 min) are compared in Figure 4. Fibers produced from Orgacon PEDOT: PSS (U, S5, F5 and D5) had lower electrical conductivities than their Clevios PH1000 counterparts. Treating the Orgacon fibers (35 µm) increased their electrical conductivities to that of the untreated Clevios fibers (20 and 90 µm). Both the diameters of Clevios PH1000 fibers (PHU and PHTU) displayed similar starting conductivities of approximately 120 S cm^−^^1^ with thin, 20 µm fibers showing a larger conductivity increase after treatment. This may be due to the fact that the removal of PSS from the surface of a thinner fiber will have a greater effect than from a thicker fiber. There was a three to four-fold increase in conductivity up to 420 ± 50 S cm^−^^1^ for thicker Clevios fibers (90 µm fibers-PHS5, PHF5, PHD5) and a six-fold increase for thin fibers up to 800 ± 120 S cm^−^^1^ (20 µm fibers-PHTS5, PHTF5, PHTD5). The 20 µm fibers achieved electrical conductivities three times higher than Jalili et al. reported for PEDOT fibers treated with ethylene glycol [9].

Average ultimate tensile strength values for all three fiber sizes are also presented in Figure 4. It can be seen that thin PHTU fibers displayed higher ultimate strengths than their thicker PHU counterparts. This increase in strength is attributed to a more controlled coagulation process when using the lower flow-rate and needle diameter for these thin, 20 µm diameter fibers. This control produced fibers with fewer variations in diameter and surface defects, as seen in the SEM images (Figure 3); and as a result a stronger fiber. Sulfuric acid treatments decreased the strength of the fibers, whilst formic acid and DMSO treatments showed no discernable change in the breaking strengths of Clevios PH1000 fibers.

**Figure 5:**
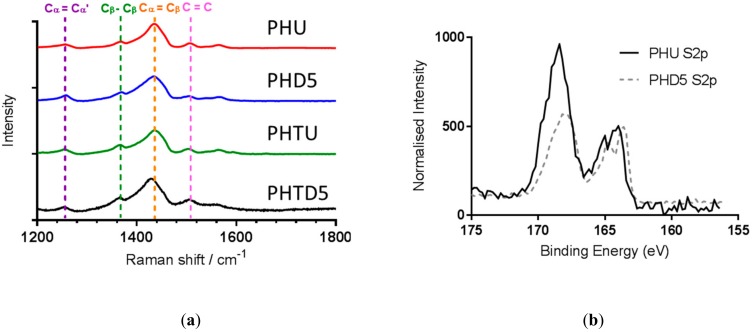
(**a**) Raman spectra for Clevios fibers untreated and treated with DMSO for 5 min. Untreated and 5 min DMSO treatments for PH1000 thick and PH1000 thin. (**b**) XPS spectra showing S2p emission for fibers PHU and PHD5.

Raman spectra of untreated and DMSO-treated Clevios PH1000 fibers are shown in Figure 5. The peaks at 1258 cm^−1^ and 1368 cm^−1^ are attributed to Cα-Cα inter-ring stretching and Cβ-Cβ stretching vibrations of PEDOT, respectively whilst the peak at 1437 cm^−1^ is due to the symmetrical stretch of Cα = Cβ quinoid form of PEDOT. The peak at 1505 cm^−1^ is due to asymmetrical C = C stretches of the PSS dopant. These correspond accordingly with previously published spectra of PEDOT [23]. The symmetrical stretch of Cα = Cβ quinoid from the PEDOT thiophene ring shifted from 1437 cm^−1^ in the untreated fibers to 1433 cm^−1^ in the 90 µm DMSO-treated fibers (PHD5) and 1429 cm^−1^ in the 20 µm DMSO-treated fibers (PHTD5). Similar shifts have been observed in Raman spectra of ethylene glycol-treated PEDOT: PSS films and fibers [9] and has been previously observed for reduced PSS dopant concentration in PEDOT: PSS [24]. This suggests that DMSO reduces the insulating PSS content in the fiber leading to the observed increase in conductivity.

To further confirm the effect of treatment on the fibers, XPS spectra were obtained to allow for comparison before and after treatment. Figure 5 shows the XPS results for the 155–175 eV range, for PHU and PHD5 fibers. This range shows the emissions from the S2p electrons from sulfur present in the fiber. Two main peaks are observed, with the peak at 162–166 eV corresponding to sulfur present in the thiophene ring of PEDOT, while the peak at 166–171 eV corresponds to sulfur present in the PSS. The spectra have been normalized to the intensity of the PEDOT sulfur peak, to allow comparison of the PSS sulfur before and after treatment with DMSO. The emission produced by PSS sulfur decreased by approx. 50% after treatment, showing a reduction in the amount of PSS present on the surface of the fiber. This agrees with the results previously reported for PEDOT: PSS treated with DMSO [25,26] and other solvents [18].

**Figure 6:**
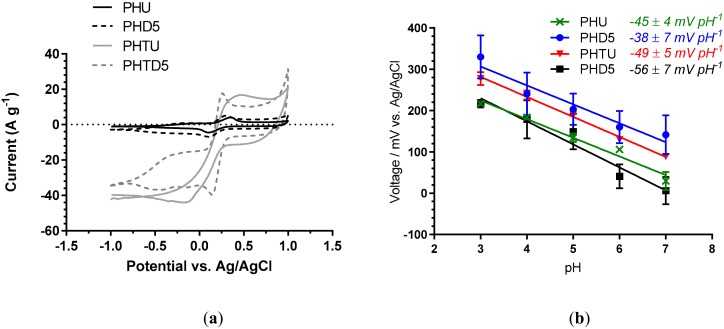
(**a**) Cyclic voltammograms of fibers PHU, PHD5, PHTU and PHTD5. Measured at a scan rate of 50 mV s^−1^ vs. Ag/AgCl electrode in 10 mmol dm^−3^ K_3_[Fe(CN)_6_] with 0.1 mol dm^−3^ KCl. (**b**) Potentiometric (open circuit) response, in buffered solutions of varying pH, of 25 and 75 µm PEDOT:PSS wet-spun fibers (without and with DMSO treatment) coated with Pani via electropolymerisation of aniline solutions (0.1 M) in nitric acid (1.0 M). Electrodeposition was via CV: −0.2 to +1.0 V vs. Ag/AgCl, 100 mV s ^−1^, 10 cycles. n = 3.

Fibers produced from Clevios PH1000 PEDOT: PSS produced more highly conducting fiber electrodes than their Orgacon counterparts, even without solvent treatment to remove insulating PSS. Both Clevios fiber sizes (20 and 90 µm diameter) were taken forward to examine their performance as fiber-based sensing electrodes. To further establish their electrochemical properties, the electrochemical surface area was examined. Cyclic voltammetry using potassium ferricyanide redox probe was performed at different scan rates to calculate the electrochemical surface area of Clevios PH1000 fibers before and after solvent treatment. A clear difference can be seen in the response observed for untreated and treated fibers (Figure 6a) while all three treatments show similar responses (data shown for DMSO treatment only). Analysis of the peak currents using the Randles-Sevcik equation show that following DMSO-treatment, 90 µm Clevios PH1000 fibers increased their electrochemically accessible surface area from 0.077 cm^2^ (PHU) to 0.095 cm^2^, 0.095 cm^2^, and 0.096 cm^2^ for PHS5, PHF5, and PHD5 fibers respectively. The 20 µm Clevios fibers increased their electrochemically accessible surface area from 0.019 cm^2^ (PHTU) to 0.034 cm^2^, 0.033 cm^2^, and 0.032 cm^2^ post treatment for PHTS5, PHTF5, and PHTF5 fibers respectively. All three treatments show similar increases in surface area, increasing to approximately 125% of the untreated value for 90 µm fibers (PHU), and approximately 175% of the untreated value for the 20 µm fibers (PHTU).

To make fiber-based sensors, Pani was chosen for deposition onto PEDOT: PSS fibers due to its pH-sensitivity. Although the 20 µm, DMSO-treated fibers achieved the highest conductivity, the pH sensing performance of both fiber diameters was explored to ensure the optimum fiber for a wearable pH sensor was identified. Pani was electrodeposited onto fibers in a similar manner reported by Vacca et al. (0.1 M aniline in 1.0 M HNO_3_) who electropolymerised Pani onto an ink-jet printed PEDOT: PSS film [27]. Following Pani deposition via CV onto the DMSO treated Clevios PH1000 fibers (PHU, PHD5, PHTU, and PHD5), the pH sensitivity was assessed against a double-junction Ag/AgCl electrode (Figure 6b). The 75 µm thick fibers gave the poorest pH sensitivity irrespective of solvent treatment: −45 ± 4 mV pH^−1^ (no treatment) and −38 ± 7 mV pH^−1^ (DMSO treated). The 25 µm thick fibers fabricated without solvent treatment showed an improved pH sensitivity of −49 ± 5 mV pH^−1^. As established by the conductivity values (Figure 4a), the thicker fibers have a higher PSS to PEDOT ratio, which leads to more water being absorbed by the hydrophilic PSS, resulting in more swelling during pH analysis (data not shown); this would affect the resistance of the electrode and thus, the potential and slope of voltage vs. pH [28]. Additionally, the 75 µm thick fibers have a lower conductivity than the 25 µm thick fibers, possibly resulting in the formation of a less effective Pani film and hence, poor pH sensitivity. The best pH response and lowest swelling was achieved by the thin fiber that underwent DMSO treatment, which gave a near Nernstian response of −56 ± 7 mV pH^−1^ (n = 3) (Figure 6b). This thin DMSO-treated fiber exhibited the highest conductivity (802 ± 122 S cm^−1^) as well as the best pH response when Pani coated. Further optimization is underway to improve the performance of these promising fiber-based pH sensors.

## 4. Conclusions

Three variants of PEDOT: PSS fibers were each treated with sulfuric acid, formic acid, and DMSO. Analysis of the consequence of these treatments on PEDOT: PSS fibers has shown the treatments to be effective in modifying many of the properties of the fibers after 5 min, with no further improvement being noted with longer treatment times. An increase in electrical conductivity was observed for formic acid and DMSO treatments without significantly changing the breaking strength of the fibers, while sulfuric acid showed an increase in electrical conductivity at the expense of tensile strength. The increase in electrical conductivity is due to a reduction of the insulating PSS within the fibers as shown by Raman spectroscopy and XPS measurements. The fiber most suited for use in electrochemical devices was produced from Clevios PH1000 with a 20 µm diameter, and treated with DMSO. These fibers possess an optimal balance of electrical conductivity and mechanical strength. Pani deposition onto this DMSO-treated 20 µm fiber resulted in a fiber-based pH sensor with a Nernstian response of −56 ± 7 mV pH^−1^. These fibers are promising potentiometric sensors for wearable applications.

## Figures and Tables

**Table 1:** Nomenclature of three fiber types spun from Orgacon and Clevios PH1000 PEDOT: PSS.

		5 Min Solvent Treatment			
PEDOT: PSS Source	Fiber Diameter (µm)	Untreated	Sulfuric Acid	Formic Acid	DMSO
Orgacon	35	U	S5	F5	D5
Clevios PH1000	90	PHU	PHS5	PHF5	PHD5
Clevios PH1000	20 * (* T = thin)	PHTU	PHTS5	PHTF5	PHTD5
